# Supplementary material for: Novel Driver Strength Index highlights important cancer genes in TCGA PanCanAtlas patients
Source: PeerJ. 2022 Aug 11;10:e13860. doi: 10.7717/peerj.13860 (PMC9375969; doi:10.7717/peerj.13860)
Supplement: Supplemental Information 2 [file peerj-10-13860-s002.zip › COHORTS/genes plots/detailed/2021_8_16_14_9_distribution_events_detailed_males_13.pdf]

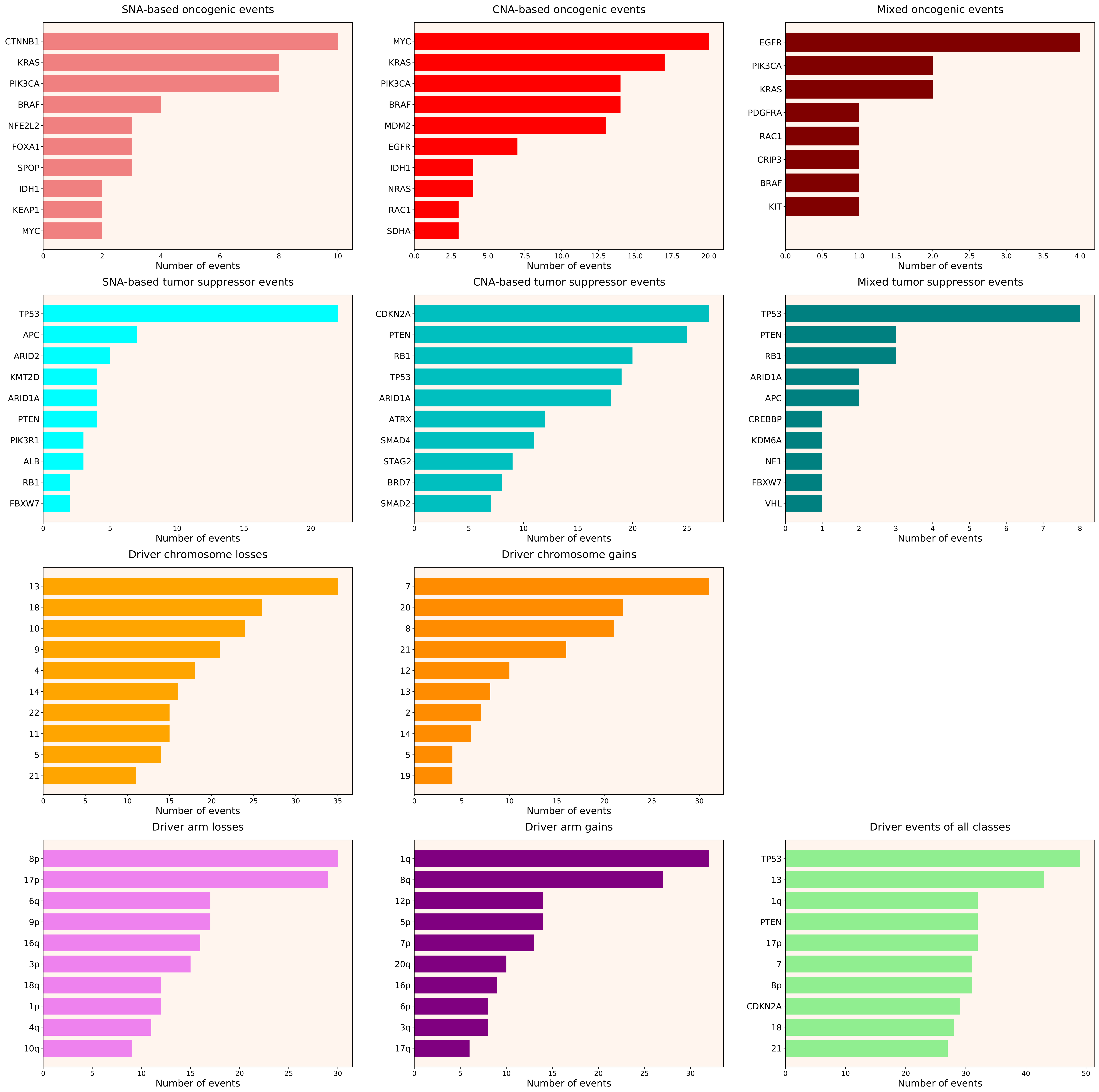
SNA-based oncogenic events
CTNNB1
KRAS
PIK3CA
BRAF
NFE2L2
FOXA1
SPOP
IDH1
KEAP1
MYC
0 2 4 6 8 10
Number of events
CNA-based oncogenic events
MYC
KRAS
PIK3CA
BRAF
MDM2
EGFR
IDH1
NRAS
RAC1
SDHA
0.0 2.5 5.0 7.5 10.0 12.5 15.0 17.5 20.0
Number of events
Mixed oncogenic events
EGFR
PIK3CA
KRAS
PDGFRA
RAC1
CRIP3
BRAF
KIT
0.0 0.5 1.0 1.5 2.0 2.5 3.0 3.5 4.0
Number of events
SNA-based tumor suppressor events
TP53
APC
ARID2
KMT2D
ARID1A
PTEN
PIK3R1
ALB
RB1
FBXW7
0 5 10 15 20
Number of events
CNA-based tumor suppressor events
CDKN2A
PTEN
RB1
TP53
ARID1A
ATRX
SMAD4
STAG2
BRD7
SMAD2
0 5 10 15 20 25
Number of events
Mixed tumor suppressor events
TP53
PTEN
RB1
ARID1A
APC
CREBBP
KDM6A
NF1
FBXW7
VHL
0 1 2 3 4 5 6 7 8
Number of events
Driver chromosome losses
13
18
10
9
4
14
22
11
5
21
0 5 10 15 20 25 30 35
Number of events
Driver chromosome gains
7
20
8
21
12
13
2
14
5
19
0 5 10 15 20 25 30
Number of events
Driver arm losses
8p
17p
6q
9p
16q
3p
18q
1p
4q
10q
0 5 10 15 20 25 30
Number of events
Driver arm gains
1q
8q
12p
5p
7p
20q
16p
6p
3q
17q
0 5 10 15 20 25 30
Number of events
Driver events of all classes
TP53
13
1q
PTEN
17p
7
8p
CDKN2A
18
21
0 10 20 30 40 50
Number of events
